# Assessment of Oral Health Knowledge, Attitude, Practice and DMFT Scores among Patients at King Faisal University, Al-Ahsa

**DOI:** 10.3390/medicina59040688

**Published:** 2023-03-30

**Authors:** Muhammad Adeel Ahmed, Rizwan Jouhar, Muhammad Faheemuddin, Ahmed AlJafar, Hussain Alabawi, Baqer Alhumaidi, Moaiad Al Madeh

**Affiliations:** 1 Department of Restorative Dental Sciences, College of Dentistry, King Faisal University, Al-Ahsa 31982, Saudi Arabia; 2 Department of Operative Dentistry and Endodontics, Altamash Institute of Dental Medicine, Karachi 75500, Pakistan; 3 Department of Prosthodontics and Implantology, College of Dentistry, King Faisal University, Al-Ahsa 31982, Saudi Arabia

**Keywords:** oral public health, DMFT index, dentistry, caries prevention, oral quality of life

## Abstract

*Background and Objectives*: Oral health is one of the most significant issues in public health. The Decayed, Missing, and Filled Teeth (DMFT) Index is a useful tool for assessing and measuring the state of oral health in a community. This study aimed to evaluate oral health knowledge, attitudes, and practices among participants who visited a dental clinic at King Faisal University and to evaluate their DMFT scores. *Materials and Methods:* This cross-sectional, questionnaire-based study was conducted at the King Faisal University dental complex, Kingdom of Saudi Arabia, using a simple random sampling technique. The data were collected using a self-administered structured questionnaire in English and Arabic. All statistical analyses were carried out using the SPSS 20 software. A chi square and ANOVA test were used to assess the association. A *p* value of <0.05 was considered statistically significant. *Results:* There were a total of 260 participants, of whom 193 (74.2%) were male and 67 (25.8%) were female. Most participants, 173 (66.5%), were between the ages of 18 and 28. The majority of the participants 191 (73.5%) believed that bad oral hygiene led to gum disease. Additionally, major issues while visiting dental clinics, the importance of routine dental clinic visits, the existence of a connection between oral and general health, brushing time and frequency of change of used brush were significantly influenced by gender (*p* < 0.05). In terms of the DMFT index, mean numbers of decaying teeth (D) were 4.82 ± 4.15, mean numbers of missing teeth (M) were 1.56 ± 2.94, mean numbers of filled teeth (F) were 5.17 ± 5.28 and mean DMFT score was 11.56 ± 6.32, with a statistically significant difference observed (*p* < 0.001). *Conclusions:* This study concluded that, although some of the study participants neglected oral hygiene practices, the majority of participants had good knowledge and attitudes regarding the significance of oral hygiene. Owing to inadequate practices, the decayed, missing, and filled teeth scores increased with increasing age. Additionally, gender had no significant impact on the mean scores for decayed, missing, and filled teeth, although there were significant differences between age groups.

## 1. Introduction

Oral health is an important aspect of public health [1], and is strongly linked to an individual’s health [2,3]. It has such an impact on human activities such as eating, talking, interpersonal interactions, and quality of life that the World Health Organization has designated oral health as one of the most widespread and serious public health issues [4].

Oral health knowledge is a necessary prerequisite for health-related behavior, and this improves with age. Individuals’ measures to protect, maintain, or restore health and to avoid sickness are referred to as health-related behaviors [5]. Oral care products are those that are used to clean the mouth, freshen the breath, and maintain oral hygiene. Because dentistry is constantly improving, a wide range of oral care products are now accessible in the market, rendering selection rather difficult. Several techniques might influence the choice of oral care products, which are important in promoting oral health and avoiding dental problems [6]. Brushes and toothpaste are the most commonly used oral hygiene products. Individuals also utilize other oral hygiene aids, either on the advice of a dental clinician or by themselves. Routine dental check-ups [7], brushing periodicity [8], proper dietary habits and less sugar intake [9], use of dental floss, and other techniques of inter-dental cleaning [10] are all necessary for maintaining good oral health. These methods are crucial in the prevention of caries and periodontal inflammation [11]. Several research have found a substantial relationship between oral health status and level of knowledge. Because the primary goal of healthcare practitioners is to promote health and provide preventative knowledge, they should establish high standards of oral health competence that adhere to professional criteria [12].

Several studies on oral health knowledge, perceptions, and practices among university students have been performed in various countries [13,14,15,16,17]. A study by Peltzer and Pengpid evaluated oral health behavior and related determinants amongst undergraduate university students in 26 poor, moderate, and high-income countries. This study revealed poor rates of brushing teeth among students from Asia, Africa, and the Americas [13]. A study was carried out in Nigeria to examine medical, pharmacy, and nursing university students’ oral health knowledge, attitudes, and behavior. The researchers discovered that students’ oral health-related knowledge, attitudes, and behavior needed to be improved [14]. Similarly, Kumar et al. investigated and contrasted oral health awareness, attitudes, and behaviors among dentistry and medical students. They discovered that females had superior oral health knowledge and habits than males [15]. Several studies have been undertaken in Arab nations to examine oral health-related knowledge, attitudes, and practices, particularly among school children [16,17].

Poor oral health encompasses a wide spectrum of oral illnesses, one of which is dental decay. Tooth decay is a serious public health problem [18,19] that affects people of all age groups, genders, ethnicities, and socioeconomic backgrounds [2]. The World Health Organization estimates that around 3.5 billion people, or 35.5% of the entire population, have irreversible dental decay [20]. Iran also has a high rate of dental decay. According to the findings of a nationwide survey in Iran between 2001 and 2002, the average tooth decay in 18 years old was 3.4, and in 35–45 years old it was 11 times greater than in the general population [4].

When bacterial pathogens colonize the tooth surface, decay occurs [18]. However reversible in the initial phases, if left untreated, it advances slowly and damages hard dental tissue. This can also result in tooth loss, causing physical and social distress in those afflicted [21]. Tooth decay is a multifaceted condition that is heavily influenced by dietary habits such as sugar consumption and individual practices [3]. Different parameters have been discovered in the frequency of dental decay in several studies carried out across the world, including age, gender, educational status, brushing, smoking, biological factors, and financial and social status [22].

The Decayed, Filled, and Missing teeth (DMFT) index is the most significant indicator used to measure oral health status [20], and it is recognized by the World Health Organization for quantifying and comparing the incidence of dental decay in a community [23]. This index measures the number of decaying, missing, and filled teeth and is utilized to assess and monitor oral health treatments, take measures, and carry out interventional programs. Because the majority of epidemiological research in this area concentrates on youngsters [18], adults have been given less consideration [20]. According to the findings of a nationwide oral health study performed in 2001 and 2002, the mean DMFT score for young individuals aged 18 was 3.4, and 11.0 for adults aged 35 to 45. Furthermore, 53% of those aged 35 to 44 developed periodontal pockets [24]. Individuals aged 15 to 45 years old are the most economically successful group in society, and poor dental health might interfere with their everyday activities. Nonetheless, this population group shows less data on the frequency of dental caries and oral health status than in children and the elderly. Therefore, the purpose of this study was to assess oral hygiene maintenance awareness, attitude, and practices and to evaluate the DMFT scores among participants of different age groups at King Faisal University.

## 2. Materials and Methods

This cross-sectional, descriptive questionnaire-based study was conducted at the King Faisal University dental complex, Eastern Province, Kingdom of Saudi Arabia, using a simple random sampling technique. Ethical approval for this study was obtained from the committee of scientific research at King Faisal University, Al-Ahsa (KFU-REC-2022-JAN-EA000346, approved on 4 January 2022). A total of 260 participants of both genders, who were aged 18 to >60 years old, attending dental clinics were included in the study. Severe medically comprised patients, incomplete questionnaires and those who did not give consent were excluded. Using Cochran’s formula for estimation of sample size, taking 50% for population at risk, 95% confidence interval and 5% margin of error, the estimated sample size was 385. However, 125 participants were excluded for several reasons as explained in the exclusion criteria. Eventually, data analysis were formed on 260 participants.

The information was gathered using a self-administered structured questionnaire in English and Arabic, depending on the patient’s preference. Following an examination of the patients participating in this research, a digital questionnaire form was distributed to them. The questionnaire consisted of 15 multiple-choice questions meant to assess the participants’ oral health knowledge, attitudes, and practices. The first part of the questionnaire covered basic information, such as age, gender, and educational status. The second part measured the participants’ oral health knowledge by asking questions concerning the cause and prevention of tooth decay, the purpose of visiting the dentist, the oral hygiene procedure used to clean the teeth, and the impact of oral health on general health. The third part was used to review students’ attitudes on the importance of regular dental appointments, the impact of soft drinks on teeth, and their attitudes toward dental treatment. The last part assessed oral health practices by asking questions about the materials used, frequency of brushing, and usage of fluoride mouthwash.

The DMFT score of the study participants was calculated based on the clinical examination findings and the number of decaying (D), filled (F), and missing (M) teeth owing to caries. Researchers collected data by observing and directly inspecting the participant’s teeth using a mouth mirror on a dental chair. The questionnaire was filled out after each subject was examined.

### Statistical Analysis

The collected data were analyzed using the Statistical Package for the Social Sciences Software (SPSS Statistics, version 25, Chicago, IL, USA). Descriptive statistics were calculated by using means and standard deviations for continuous variables and frequencies and percentages for categorical variables. The Shapiro–Wilk test was applied to assess the normality of the data. The chi-square test was applied to explore the influence of gender and age group on oral hygiene knowledge, attitudes and practices. Using ANOVA (Scheffé post hoc), the DMFT index was assessed for different age groups and genders. A *p*-value of ≤0.05 was considered statistically significant.

## 3. Results

A total of 260 participants took part in this study. Of all the participants, 193 (74.2%) were male and 67 (25.8%) were female; 173 (66.5%) of the participants were between the ages of 18 and 28; 39(15.0%) were between the ages of 29 and 40; 21 (8.1%) were between the ages of 41 and 50; 15 (5.7%) were between the ages of 51 and 60; and 12 (4.6%) were beyond the age of 60. According to the distribution of participants in the study by educational level, 146 (56.2%) had college degrees, 15 (5.7%) had completed only primary school, 22 (8.5%) had up to middle school education, 5 (1.9%) had a diploma, and 72 (27.7%) had completed only high school, as shown in Table 1.

Regarding knowledge, attitude and practices for oral hygiene, the majority of participants, 191 (73.5%), agreed that poor oral hygiene causes gum disease, whereas just 16 (6.2%) disagreed, and 53 (20.4%) were unaware. The majority of participants 150 (57.7%) thought that using dental floss, brushing with paste, and taking vitamin C supplements might help prevent gum disease. However, only 20 (7.7%) individuals thought that using dental floss, and 55 (21.2%) believed that taking vitamin C supplements could prevent gum disease, whereas 34 (13.1%) showed no knowledge. The majority of participants, 151 (58.1%), preferred to go to the dentist when they experienced pain, while 54 (20.8%) preferred to go every six months, 24 (9.2%) visited every 3 months, and 28 (10.8%) visited every year. Furthermore, 136 (52.3%) participants assumed that treatment cost was the major concern in dental treatment, and 73 (28.1%) experienced dental fear. Routine visits to the dental clinic are vital, according to the majority of participants, 201 (77.3%), whereas 20 (7.5%) disagree. However, 39 (15.0%) participants were unaware of the significance of dental appointments. Around 188 (72.3%) participants were aware that there is a link between oral and general health; 56 (21.5%) participants were unaware of the relationship; and 16 (6.2%) participants were of the opinion that there is no link. It is interesting to note that 228 (87.7%) participants agreed that cleaning teeth helps prevent dental caries. The majority of participants, 243 (93.5%), agreed that eating a lot of sugar causes dental decay, and 218 (83.3%) participants overwhelmingly agreed that soda and other carbonated beverages cause tooth decay. About 152 (58.5%) participants agreed that using fluoride toothpaste reduced the likelihood of tooth decay, while 88 (33.8%) participants were unaware of this. Among the participants, 71 (27.3%) brushed their teeth once daily, 79 (30.4%) brushed twice daily, 23 (8.8%) brushed three times daily, and 87 (33.5%) did not brush their teeth at all. According to the participants’ responses regarding their knowledge of interdental cleaning aids, the majority of participants, 123 (47.3%), used only a brush with toothpaste for interdental cleaning, while 120 (46.2%) preferred dental floss, or miswak, in combination with a brush with tooth paste for interdental cleaning. The majority of participants, 78 (30.0%), brush their teeth before going to sleep and after waking up, 58 (22.3%) clean their teeth before going to sleep, and 67 (25.8%) do not brush regularly. Almost 95 (36.5%) of research participants replaced their brush every three months, and 89 (34.2%) changed their brush once a year. Ideally, the brush should be replaced every 3 to 4 months. The majority of participants, 181 (69.6%), do not use antimicrobial mouthwash, whereas 47 (18.1%) use it 2–3 times per week, as shown in Table 2.

The association of age groups with knowledge, attitude, and practices of oral hygiene revealed that, among the different age groups of study participants, 143 (82.7%) of 18–28 years, and 29 (74.4%) of 29–40 years, had knowledge of how poor oral hygiene leads to gum disease, and the difference is statistically significant (*p* < 0.001). Furthermore, 110 (63.6%) of 18–28 year old participants utilized dental floss, toothbrush with paste, and Vitamin C to avoid gum disease, with a statistically significant difference noticed among all age groups (*p* < 0.001). Furthermore, 86 (49.7%) of 18–28 year old participants visited the dentist when they were in pain, whereas 45 (26.0%) visited every 6 months, with a statistically insignificant difference observed among all age groups (*p* = 0.062). More than half of the participants, 90 (52.0%) of 18–28 year olds, responded that treatment expense is the most important issue, and 54 (31.2%) reported dental fear, with a statistically insignificant difference observed among all age groups (*p* = 0.111). Furthermore, all age groups were significantly influenced by the existence of a link between oral and general health, that teeth brushing prevents dental caries, that high sugar diet and soda and other carbonated drinks lead to teeth decay, that fluoridated toothpaste decreases the chance of teeth decay, the importance of brushing frequency, and changing their brush (*p* < 0.05). On the other hand, there was an insignificant difference in usage of antibacterial mouth wash among all age groups (*p* > 0.05), as shown in Table 3.

The association of gender with oral hygiene knowledge, attitude, and practices indicated that 38 (56.7%) of females and 153 (79.3%) of males had a perspective that poor oral hygiene can lead to gum disease, with a statistically significant difference between them (*p* = 0.001). Additionally, major issues while visiting dental clinics, the importance of routine dental clinic visits, the existence of a connection between oral and general health, brushing time and frequency of change of used brush were significantly influenced by gender (*p* < 0.05), whereas the technique used to avoid gum disease, regularity of dental visits, knowledge that brushing prevents dental caries and that sugar diet and soda and other carbonated drinks lead to teeth decay, use of fluoridated toothpaste, interdental cleaning aids, and antibacterial mouthwash, and brushing frequency were insignificantly influenced by gender (*p* > 0.05), as shown in Table 4.

**Figure 1:**
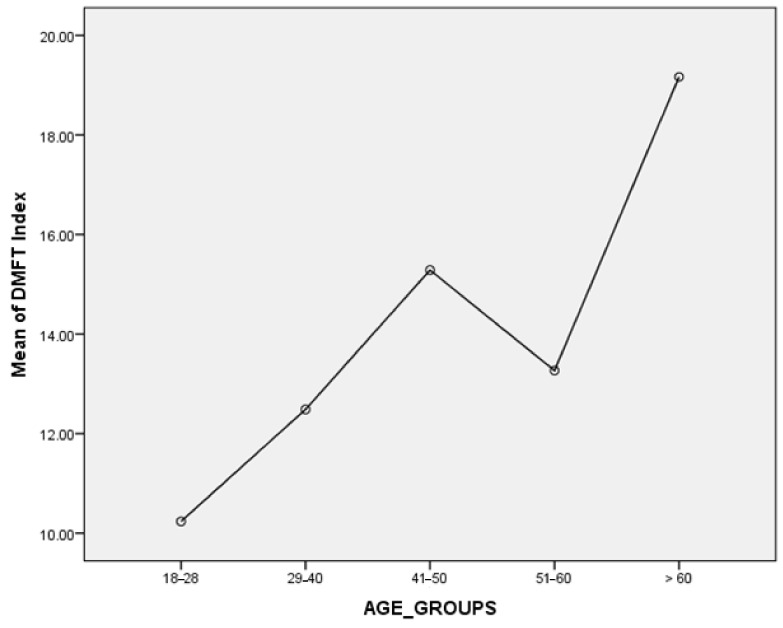
Association of mean of DMFT scores with respect to age groups.

In terms of the DMFT index, mean decaying teeth (D) were 4.82 ± 4.15, mean missing teeth (M) were 1.56 ± 2.94, mean filled teeth (F) were 5.17± 5.28 and mean DMFT score was 11.56 ± 6.32, with statistically significant difference observed (*p* < 0.001). The mean DMFT score in males was 11.41 ± 6.3 and in females 12.0 ± 6.41, with no statistically significant difference (*p* = 0.519). The mean value for total DMFT scores was 11.56 ± 6.32 in all participants, 10.23 ± 5.8 in people aged 18–28 years old, 12.48 ± 5.3 in people aged 29 to 40 years old, 15.28 ± 6.2 in people aged 41–50 years, 13.26 ± 5.9 in aged 51–60 years old and 19.1 ± 8.18 in people aged more than 60 years old, with statistically significant difference (*p* < 0.001), as shown in Table 5 and Figure 1.

## 4. Discussion

Oral disease is quite prevalent in the populations of underdeveloped nations. This may be the result of negligence, a lack of resources, inadequate education, or unfavorable attitudes. This cross-sectional study aimed to assess the knowledge, attitude and practices towards oral hygiene maintenance, as well as DMFT scores, among those who attended a dental clinic at King Faisal University.

Females are more educated than males about bleeding as an indication of periodontal disease and the need for flossing as a preventative measure. Despite the fact that there were fewer females in the present study, it became clear that they perceived oral hygiene practices with better knowledge than males did. These findings were consistent with the results of former studies, in which females had better oral health practices than males in terms of how often they brushed their teeth and visited the dentist [13,25,26]. Females often have a high concern for their appearance, which may help to explain their excellent oral health practices and attitudes. As a result, they would be more inclined to visit the dentist and learn about dental health. Regular dental visits help patients learn about oral health issues, encouraging them to practice excellent oral hygiene practices and avoid oral diseases [14,27,28].

Prolonged exposure to dental plaque could lead to gingivitis and periodontitis. Dental plaque typically accumulates on the interdental surfaces of teeth. Caries and periodontitis affect these tooth surfaces more frequently than facial areas, because they are harder to see and cannot be effectively cleaned by toothbrush filaments [29]. Because interdental cleaning helps to limit the spread and severity of both caries and periodontal disease, thorough interdental cleaning must be a primary objective of daily oral care [30]. The present study showed that about 79 (30.4%) participants brushed their teeth twice a day, 123 (47.3%) participants used a toothbrush with tooth paste for interdental cleaning and 6 (2.3%) of participants used dental floss. These findings were similar to the findings by Dali et al., in which 33% of participants reported twice-daily tooth brushing [31]. Only 2.5% of individuals used dental floss, while 84.5% of the population did not use any interdental aids. Similarly, the results of present study were corroborated by another study, wherein only 27.5% of participants brushed their teeth twice daily, [32] contrary to studies by Jiang et al. [33] and Al- Shammari et al. [34] where 67% of Chinese urban teenagers and 62% of Kuwaiti adults, respectively, brushed twice daily.

Likewise, in a study by Chakraborthy et al., very few participants utilized dental floss for interdental cleaning [35] which is comparable to our findings. Interdental aids are typically used extremely seldom as a prophylactic measure. The present study revealed that participants were unaware of simple methods for maintaining their oral health, such as tongue cleaning.

The present study revealed that about 151 (58.1%) patients visited dental clinics when they felt pain. These findings were in accordance with another study, in which 54% of participants had only visited dentists if they had toothache or dental issues [36].

The present study found that the majority of the 188 (72.3%) participants were aware of the existence of a link between oral and general health. These findings were supported by another study, which found that, while around 60% of people were aware of the link between oral health and general health, their oral hygiene maintenance practices were inadequate [32]. Similarly, our findings differed from those of Kapoor et al. [37] and Sen et al. [38], in that a greater number of participants were unaware that oral health is linked to general health.

According to the findings of the present study, about 123 (47.3%) participants used a brush with tooth paste to clean their teeth, compared to 1 miswak (0.4%), 6 floss (2.3%), and 120 (46.2%) participants used a brush with tooth paste in conjunction with dental floss, miswak, and toothpicks. These findings were partially consistent with the findings of a previous study, which found that more than 60% of respondents used toothpaste and a toothbrush to clean their teeth, as opposed to toothpaste and a toothbrush combined with floss, miswak, no brushing, or other products [39]. In contrast, Hussain et al. revealed that the majority of subjects (88%) used a toothbrush to clean their teeth, 5.5% used toothpowder, 5% used their finger, and 1.5% did not use any technique at all [32].

Concerning brushing frequency, the present study showed that 57 (29.5%) of males and 14 (20.9%) of females brushed their teeth once a day, and 57 (29.5%) of males and 20 (29.9%) of females brushed their teeth twice a day. These findings contradicted previous research, which found that 33% of males and 34% of females brushed their teeth once daily [39], whereas Hussain et al. discovered that 65% of participants brushed only once a day, 27.5% brushed twice a day, 3.5% brushed their teeth sometimes, and only 4% brushed more than twice daily [32].

Another piece of research on oral hygiene awareness, attitudes, practices and overall dental appearance among female students, conducted by Elsabagh et al., found that 42.4% brushed twice daily, with 29% brushing for 2 min. These findings were comparable to those found in an Indian study, where 50.4% of respondents reported brushing their teeth twice daily, and 83.9% did not use dental floss [40]. The present study’s findings contradicted those of previous studies, revealing that 79 (30.4%) of participants brushed twice daily, but only 6 (2.3%) used dental floss.

The present study found that the majority of participants were knowledgeable about oral health. A majority of 191 (73.5%) participants knew that poor oral hygiene causes gum disease. These findings were consistent with previous research, which found that pupils had an excellent understanding of oral health. The majority of students (63%) were aware that poor oral health leads to gum disease. To pursue healthy oral habits, it is vital to have a thorough knowledge of oral health [41]. Increased knowledge was linked to better oral health in studies done in Kuwait and Spain [42,43]. However, good dental health not only helps an individual look and feel well, but it also aids in the preservation of oral functions [44].

The Decayed, Missing, and Filled Teeth (DMFT) index is a useful tool for measuring and monitoring a community’s oral health status. The present study showed that the mean for decayed teeth among the participants was 4.82 ± 4.15, mean missing teeth was 1.56 ± 2.94, and mean filled teeth was 5.17 ± 5.28. Moreover, the mean DMFT score was observed as 11.56 ± 6.32. These findings were partially consistent with a previous study, which found that the participants’ mean values for Decayed teeth (DT), Missing teeth (MT), and Filled teeth (FT) were 2.85 ± 1.7, 1.15 ± 1.84, and 3.33 ± 1.7, respectively. The mean value of the total DMFT index was 7.33 ± 3.0 for all participants, 6.9 for individuals aged 15 to 19, and 7.8 for persons aged 35 to 45 [4]. The lack of family attention to oral health, poor financial accessibility caused by the lack of coverage for such treatments in insurance plans, and the lack of government support for community-based oral health promotion initiatives are likely to be the causes.

Similarly, the present study showed that the mean DMFT score was 11.56 ± 6.32, with a statistically significant difference found between the mean DMFT and age group (*p* < 0.001). The mean DMFT was high in the age groups 18–20, 29–40, and >60 years, and low in age groups 41–50 and 51–60. The comparatively low DMFT score in the age group 41–60 may be link to the participant’s education and socioeconomic status. These findings were corroborated by previous studies. In Japan the mean DMFT index was 12.2, in Malaysia, it was 12.1, and in Turkey 10.8 [45]. On the contrary, these findings were not supported by another study that found the mean DMF score in all participants was 18.06 ± 8.7. Despite the fact that there was no statistically significant difference between the mean DMF in the age groups 40 to 50, 50 to 60, and 60 to 70, it is important to keep in mind that the population sizes of the three age groups were not similar, with people aged 61 to 70 making up about half of all age groups [46], although younger people are more likely to develop dental decay because they consume more sweets and less natural dairy items.

The findings of the study must be seen in the light of certain limitations. First, due to the cross-sectional nature of this study, it is possible that the cause-and-effect link between some of the study’s variables cannot be accurately determined. Second, uneven gender distribution may not truly represent the study area population. Third, our study was also restricted to a particular region with unique cultural, economic, and social conditions. Fourth, additional preventive treatment such as fluoride, casein phospho-peptide-amorphous calcium phosphate and biomimetic hydroxyapatite, which have shown a promising results in DMFT reduction and caries prevention, were not taken into consideration [47,48,49]. Finally, although the DMFT index is the most commonly used index globally, it has certain drawbacks. The F or M component could show various problems in addition to previously decaying teeth that are unrelated to dental caries. Therefore, unless the target population performs it optimally, awareness alone is insufficient to promote good oral health. Even though spreading awareness about oral health starts with education, monitoring how well it is being implemented is a key sign of how well the message is getting across.

Future prospective studies are desirable to thoroughly implement and evaluate the effects of oral hygiene education and practices on DMFT scores, while keeping socioeconomical factors in mind.

## 5. Conclusions

Although some of the study participants neglected oral hygiene practices, the majority of participants had good knowledge and attitudes regarding the significance of oral hygiene. Owing to inadequate practices, decayed, missing, and filled teeth score increased with increasing age. Consequently, improving socioeconomic status, raising parents’ and children’s levels of education, modifying behavioral routines (such as brushing, mouthwash use, and dental floss), and expanding access to health insurance are all ways to enhance oral and dental health.

## Figures and Tables

**Table 1:** Demographic details of study participants (*n* = 260).

Variable		*n*	(%)
**Age (years)**	**18–28**	173	66.5
	**29–40**	39	15.0
	**41–50**	21	8.1
	**51–60**	15	5.8
	**60 and Above**	12	4.6
**Gender**	**Male**	193	74.2
	**Female**	67	25.8
**Education level**	**Primary School**	15	5.8
	**Mid school**	22	8.5
	**College education**	146	56.2
	**Diploma**	5	1.9
	**High school**	72	27.7

**Table 2:** Prevalence of knowledge, attitude and practices towards oral health maintenance of study participants.

Variables		*n*	(%)
**Does bad oral hygiene lead to gum disease?**			
	**Yes**	191	73.5
	**No**	16	6.2
	**I don’t know**	53	20.4
**How do you avoid gum disease?**			
	**Using dental floss**	20	7.7
	**Using toothbrush with paste**	55	21.2
	**Using vitamin C**	1	0.4
	**Combination of the above**	150	57.7
	**I don’t know**	34	13.1
**How often do you visit your dentist?**			
	**Every 3 months**	24	9.2
	**Every 6 months**	54	20.8
	**Every year**	28	10.8
	**Never visited a dentist**	3	1.2
	**When I feel pain only**	151	58.1
**What is your major concerns while visiting dental clinics?**			
	**Dental clinics nearby**	51	19.6
	**Dental phobia**	73	28.1
	**Treatment cost**	136	52.3
**Does routine visits to the dental clinic are important?**			
	**Yes**	201	77.3
	**No**	20	7.7
	**I don’t know**	39	15.0
**Is oral health and general health are connected?**			
	**Yes**	188	72.3
	**No**	16	6.2
	**I don’t know**	56	21.5
**Does Teeth brushing prevent dental caries?**			
	**Yes**	228	87.7
	**No**	15	5.8
	**I don’t know**	17	6.5
**Does high sugar diet lead to tooth decay?**			
	**Yes**	243	93.5
	**No**	3	1.2
	**I don’t know**	14	5.4
**Does Soda and other carbonated drinks lead to teeth decay?**			
	**Yes**	218	83.8
	**No**	14	5.4
	**I don’t know**	28	10.8
**Does fluoridated toothpaste decrease chance of the teeth decay?**			
	**Yes**	152	58.5
	**No**	20	7.7
	**I don’t know**	88	33.8
**How often do you brush your teeth each day?**			
	**Once daily**	71	27.3
	**Twice daily**	79	30.4
	**3 times a day**	23	8.8
	**I do not brush teeth regularly**	87	33.5
**Which cleaning aids do you use?**			
	**Brush with toothpaste**	123	47.3
	**Dental floss**	6	2.3
	**Miswak**	1	0.4
	**Toothpicks**	3	1.2
	**Combination of the above**	120	46.2
	**I do not use any interdental cleaning aids**	7	2.7
**At what time do you usually brush your teeth?**			
	**After eating**	24	9.2
	**After waking up**	33	12.7
	**Before going to sleep and after waking up**	78	30.0
	**Before going to sleep**	58	22.3
	**I do not brush my teeth regularly**	67	25.8
**How often do you change your toothbrush?**			
	**Every 3 months**	95	36.5
	**Every 6 months**	76	29.2
	**Every year**	89	34.2
**Do you use antibacterial mouthwash?**			
	**Every day**	2	0.8
	**Every week**	30	11.5
	**2–3 times per week**	47	18.1
	**No**	181	69.6

**Table 3:** Association of knowledge, attitude and practices towards oral health maintenance with respect to age groups.

Variable		Age Groups					*p*-Value
		18–28 *n* (%)	29–40 *n* (%)	41–50 *n* (%)	51–60 *n* (%)	>60 *n* (%)	
**Does bad oral hygiene lead to gum disease?**							
	**Yes**	143 (82.7%)	29 (74.4%)	10 (47.6%)	6 (40.0%)	3 (25.0%)	<0.001
	**No**	9 (5.2%)	1 (2.6%)	3 (14.3%)	3 (20.0%)	0 (0.0%)	
	**I don’t know**	21 (12.1%)	9 (23.1%)	8 (38.1%)	6 (40.0%)	9 (75.0%)	
**How do you avoid gum disease?**							
	**Using dental floss**	15 (8.7%)	5 (12.8%)	0 (0.0%)	0 (0.0%)	0 (0.0%)	<0.001
	**Using toothbrush with paste**	28 (16.2%)	13 (33.3%)	4 (19.0%)	7 (46.7%)	3 (25.0%)	
	**Using vitamin C**	1 (0.6%)	0 (0.0%)	0 (0.0%)	0 (0.0%)	0 (0.0%)	
	**Combination of the above**	110 (63.6%)	19 (48.7%)	14 (66.7%)	5 (33.3%)	2 (16.7%)	
	**I don’t know**	19 (11.0%)	2 (5.1%)	3 (14.3%)	3 (20.0%)	7 (58.3%)	
**How often do you visit your dentist?**							
	**Every 3 months**	21 (12.1%)	2 (5.1%)	0 (0.0%)	1 (6.7%)	0 (0.0%)	0.062
	**Every 6 months**	45 (26.0%)	3 (7.7%)	3 (14.3%)	1 (6.7%)	2 (16.7%)	
	**Every year**	18 (10.4%)	3 (7.7%)	5 (23.8%)	1 (6.7%)	1 (8.3%)	
	**Never visited a dentist**	3 (1.7%)	0 (0.0%)	0 (0.0%)	0 (0.0%)	0 (0.0%)	
	**When I feel pain only**	86 (49.7%)	31 (79.5%)	13 (61.9%)	12 (80.0)	9 (75.0%)	
**What is your major concerns while visiting dental clinics?**							
	**Dental clinics nearby**	29 (16.8%)	6 (15.4%)	8 (38.1%)	4 (26.7%)	4 (33.3%)	0.111
	**Dental phobia**	54 (31.2%)	13 (33.3%)	4 (19.0%)	1 (6.7%)	1 (8.3%)	
	**Treatment cost**	90 (52.0%)	20 (51.3%)	9 (42.9%)	10 (66.7)	7 (58.3%)	
**Does routine visits to the dental clinic are important?**							
	**Yes**	149 (86.1%)	30 (76.9%)	12 (57.1%)	7 (46.7%)	3 (25.0%)	<0.001
	**No**	13 (7.5%)	3 (7.7%)	2 (9.5%)	1 (6.7%)	1 (8.3%)	
	**I don’t know**	11 (6.4%)	6 (15.4%)	7 (33.3%)	7 (46.7%)	8 (66.7%)	
**Is oral health and general health are connected?**							
	**Yes**	137 (79.2%)	31 (79.5%)	9 (42.9%)	7 (46.7%)	4 (33.3%)	<0.001
	**No**	9 (5.2%)	1 (2.6%)	1 (4.8%)	3 (20.0%)	2 (16.7%)	
	**I don’t know**	27 (15.6%)	7 (17.9%)	11 (52.4%)	5 (33.3%)	6 (50.0%)	
**Teeth brushing prevents dental caries**							
	**Yes**	156 (90.2%)	35 (89.7%)	18 (85.7%)	13 (86.7%)	6 (50.0%)	<0.001
	**No**	12 (6.9%)	2 (5.1%)	0 (0.0%)	0 (0.0%)	1 (8.3%)	
	**I don’t know**	5 (2.9%)	2 (5.1%)	3 (14.3%)	2 (13.3%)	5 (41.7%)	
**Does high sugar diet lead to tooth decay?**							
	**Yes**	164 (94.8%)	39 (100.0%)	18 (85.7%)	13 (86.7)	9 (75.0%)	0.026
	**No**	2 (1.2%)	0 (0.0%)	0 (0.0%)	0 (0.0%)	1 (8.3%)	
	**I don’t know**	7 (4.0%)	0 (0.0%)	3 (14.3%)	2 (13.3%)	2 (16.7%)	
**Does Soda and other carbonated drinks lead to teeth decay?**							
	**Yes**	152 (87.9%)	36 (92.3%)	18 (85.7%)	8 (53.3%)	4 (33.3%)	<0.001
	**No**	7 (4.0%)	1 (2.6%)	1 (4.8%)	3 (20.0%)	2 (16.7%)	
	**I don’t know**	14 (8.1%)	2 (5.1%)	2 (9.5%)	4 (26.7%)	6 (50.0%)	
**Does fluoridated toothpaste decrease chance of the teeth decay?**							
	**Yes**	113 (65.3%)	22 (56.4%)	9 (42.9%)	6 (40.0%)	2 (16.7%)	<0.001
	**No**	7 (4.0%)	2 (5.1%)	4 (19.0%)	4 (26.7%)	3 (25.0%)	
	**I don’t know**	53 (30.6%)	15 (38.5%)	8 (38.1%)	5 (33.3%)	7 (58.3%)	
**How often do you brush your teeth each day?**							
	**Once daily**	52 (30.1%)	13 (33.3%)	2 (9.5%)	3 (20.0%)	1 (8.3%)	<0.001
	**Twice daily**	64 (37.0%)	7 (17.9%)	5 (23.8%)	2 (13.3%)	1 (8.3%)	
	**3 times a day**	18 (10.4%)	3 (7.7%)	1 (4.8%)	1 (6.7%)	0 (0.0%)	
	**I do not brush my teeth regularly**	39 (22.5%)	16 (41.0%)	13 (61.9%)	9 (60.0%)	10 (83.3%)	
**Which cleaning aids do you use?**							
	**Brush with toothpaste**	80 (46.2%)	19 (48.7%)	9 (42.9%)	10 (66.7%)	5 (41.7%)	<0.001
	**Dental floss**	3 (1.7%)	0 (0.0%)	0 (0.0%)	2 (13.3%)	1 (8.3%)	
	**Miswak**	0 (0.0%)	0 (0.0%)	1 (4.8%)	0 (0.0%)	0 (0.0%)	
	**Toothpicks**	2 (1.2%)	1 (2.6%)	0 (0.0%)	0 (0.0%)	0 (0.0%)	
	**Combination of the above**	86 (49.7%)	18 (46.2%)	10 (47.6%)	3 (20.0%)	3 (25.0%)	
	**I do not use any interdental cleaning aids**	2 (1.2%)	1 (2.6%)	1 (4.8%)	0 (0.0%)	3 (25.0%)	
**At what time do you usually brush your teeth?**							
	**After eating**	17 (9.8%)	2 (5.1%)	1 (4.8%)	3 (20.0%)	1 (8.3%)	<0.001
	**After waking up**	22 (12.7%)	9 (23.1%)	0 (0.0%)	1 (6.7%)	1 (8.3%)	
	**Before going to sleep and after waking up**	60 (34.7%)	9 (23.1%)	7 (33.3%)	1 (6.7%)	1 (8.3%)	
	**Before going to sleep**	46 (26.6%)	7 (17.9%)	2 (9.5%)	2 (13.3%)	1 (8.3%)	
	**I do not brush my teeth regularly**	28 (16.2%)	12 (30.8%)	11 (52.4%)	8 (53.3%)	8 (66.7%)	
**How often do you change your toothbrush?**							
	**every 3 months**	74 (42.8%)	14 (35.9%)	3 (14.3%)	4 (26.7%)	2 (16.7%)	<0.001
	**every 6 months**	55 (31.8%)	11 (28.2%)	5 (23.8%)	3 (20.0%)	0 (0.0%)	
	**every year**	44 (25.4%)	14 (35.9%)	13 (61.9%)	8 (53.3%)	10 (83.3%)	
**Do you use antibacterial mouthwash?**							
	**every day**	2 (1.2%)	0 (0.0%)	0 (0.0%)	0 (0.0%)	0 (0.0%)	0.808
	**every week**	23 (13.3%)	4 (10.3%)	2 (9.5%)	0 (0.0%)	1 (8.3%)	
	**2–3 times per week**	36 (20.8%)	5 (12.8%)	3 (14.3%)	2 (13.3%)	1 (8.3%)	
	**No**	112 (64.7%)	30 (76.9%)	16 (76.2%)	13 (86.7%)	10 (83.3%)	

**Table 4:** Association of knowledge, attitude and practices towards oral health maintenance with respect to gender.

Variable		Male *n*(%)	Female *n*(%)	*p*-Value
**Does bad oral hygiene lead to gum disease?**				
	**Yes**	153 (79.3%)	38 (56.7%)	0.001
	**No**	11 (5.7%)	5 (7.5%)	
	**I don’t know**	29 (15.0%)	24 (35.8%)	
**How do you avoid gum disease?**				
	**Using toothbrush with paste**	45 (23.3%)	10 (14.9%)	0.328
	**Using vitamin C**	1 (0.5%)	0 (0.0%)	
	**Using dental floss**	17 (8.8%)	3 (4.5%)	
	**Combination of the above**	105 (54.4%)	45 (67.2%)	
	**I don’t know**	25 (13.0%)	9 (13.4%)	
**How often do you visit your dentist?**				
	**Every 3 months**	19 (9.8%)	5 (7.5%)	0.591
	**Every 6 months**	44 (22.8%)	10 (14.9%)	
	**Every year**	19 (9.8%)	9 (13.4%)	
	**Never visited a dentist**	2 (1.0%)	1 (1.5%)	
	**When I feel pain only**	109 (56.5%)	42 (62.7%)	
**What is your major concerns while visiting dental clinics?**				
	**Dental clinics nearby**	37 (19.2%)	14 (20.9%)	0.018
	**Dental phobia**	46 (23.8%)	27 (40.3%)	
	**Treatment cost**	110 (57.0%)	26 (38.8%)	
**Does routine visits to the dental clinic are important?**				
	**Yes**	159 (82.4%)	42 (62.7%)	<0.001
	**No**	15 (7.8%)	5 (7.5%)	
	**I don’t know**	19 (9.8%)	20 (29.9%)	
**Is oral health and general health are connected?**				
	**Yes**	147 (76.2%)	41 (61.2%)	0.012
	**No**	13 (6.7%)	3 (4.5%)	
	**I don’t know**	33 (17.1%)	23 (34.3%)	
**teeth brushing prevents dental caries**				
	**Yes**	169 (87.6%)	59 (88.1%)	0.827
	**No**	12 (6.2%)	3 (4.5%)	
	**I don’t know**	12 (6.2%)	5 (7.5%)	
**Does high sugar diet lead to tooth decay?**				
	**Yes**	178 (92.2%)	65 (97.0%)	0.253
	**No**	2 (1.0%)	1 (1.5%)	
	**I don’t know**	13 (6.7%)	1 (1.5%)	
**Does Soda and other carbonated drinks lead to teeth decay?**				
	**Yes**	163 (84.5%)	55 (82.1%)	0.682
	**No**	11 (5.7%)	3 (4.5%)	
	**I don’t know**	19 (9.8%)	9 (13.4%)	
**Does fluoridated toothpaste decrease chance of the teeth decay?**				
	**Yes**	116 (60.1%)	36 (53.7%)	0.512
	**No**	13 (6.7%)	7 (10.4%)	
	**I don’t know**	64 (33.2%)	24 (35.8%)	
**How often do you brush your teeth each day?**				
	**Once daily**	57 (29.5%)	14 (20.9%)	0.186
	**Twice daily**	59 (30.6%)	20 (29.9%)	
	**3 times a day**	19 (9.8%)	4 (6.0%)	
	**I do not brush my teeth regularly**	58 (30.1%)	29 (43.3%)	
**Which cleaning aids do you use?**				
	**Brush with toothpaste**	87 (45.1%)	36 (53.7%)	0.823
	**Toothpicks**	2 (1.0%)	1 (1.5%)	
	**Dental floss**	5 (2.6%)	1 (1.5%)	
	**Miswak**	1 (0.5%)	0 (0.0%)	
	**Combination of the above**	93 (48.2%)	27 (40.3%)	
	**I do not use any cleaning aids**	5 (2.6%)	2 (3.0%)	
**At what time do you usually brush your teeth?**				
	**After eating**	22 (11.4%)	2 (3.0%)	0.008
	**After waking up**	22 (11.4%)	11 (16.4%)	
	**Before going to sleep and after waking up**	57 (29.5%)	21 (31.3%)	
	**Before going to sleep**	50 (25.9%)	8 (11.9%)	
	**I do not brush my teeth regularly**	42 (21.8%)	25 (37.3%)	
**How often do you change your toothbrush?**				
	**Every 3 months**	78 (40.4%)	17 (25.4%)	0.049
	**Every 6 months**	56 (29.0%)	20 (29.9%)	
	**Every year**	59 (30.6%)	30 (44.8%)	
**Do you use antibacterial mouthwash?**				
	**Every day**	2 (1.0%)	0 (0.0%)	0.437
	**Every week**	20 (10.4%)	10 (14.9%)	
	**2–3 times per week**	38 (19.7%)	9 (13.4%)	
	**No**	133 (68.9%)	48 (71.6%)	

**Table 5:** The overall mean of decayed teeth, filled teeth, missing teeth and DMFT index and its association with age groups and gender.

Variables			Mean ± SD *n* (%)		*p*-Value
**Decayed teeth (D)**			4.82 ± 4.15		<0.001
**Missing teeth (M)**			1.56 ± 2.94		
**Filled teeth (F)**			5.17± 5.28		
**DMFT Index**			11.56 ± 6.32		
**DMFT** **Index**	**Gender**	**Male**	193 (74.2%)	11.41 ± 6.3	0.519
		**Female**	67 (25.8%)	12.0 ± 6.41	
	**Age groups** **(years)**	**18–28**	173 (66.5%)	10.23 ± 5.8	<0.001
		**29–40**	39 (15.0%)	12.48 ± 5.3	
		**41–50**	21 (8.07%)	15.28 ± 6.2	
		**51–60**	15 (5.76%)	13.26 ± 5.9	
		**>60**	12 (4.6%)	19.1 ± 8.18	

## Data Availability

The data presented in this study are available on request from the corresponding author. The data are not publicly available due to ethical concerns.
